# Combinatorial Resistance: The Best Defense is a Good Offense

**DOI:** 10.3389/fonc.2013.00280

**Published:** 2013-11-13

**Authors:** Kevin J. O’Donovan

**Affiliations:** ^1^Department of Chemistry and Life Sciences, United States Military Academy, West Point, NY, USA; ^2^Burke Medical Research Institute, Weill Cornell Medical College, White Plains, NY, USA

**Keywords:** BRAFV600E, vemurafenib, combination therapy, resistance mechanisms, melanoma, MAP kinase signaling system, RASopathies

In drug development, specificity and the avoidance of off-target effects are the initial holy grails, with the ultimate goal of obtaining maximal *in vivo* efficacy coupled with minimal toxicity. Tumors, however, can be very efficient at nullifying the benefits of what could otherwise be good drugs. Tumors achieve these effects through a variety of mechanisms, one of which is drug resistance.

Acquired resistance to cancer therapies remains a major health problem in oncology (1). Targeted therapies inhibit tumor growth but success is often short-lived as tumors rapidly evolve a way to bypass the pharmacologic block via re-activation of the targeted or parallel pathways. So, what can be done to overcome acquired resistance and achieve desired outcomes in the clinic? Perhaps the best defense against acquired resistance is to come out swinging and deal with it up front, rather than waiting for the inevitable to occur and subsequently modifying treatment regimens on the fly.

Two recent papers (2, 3) shed light on a novel combination therapy strategy to circumvent acquired resistance in BRAF^V600E^-positive melanomas. One is a mechanistic study (3) that shows why combination therapy may be promising for BRAF^V600E^-positive melanomas and that it can work in xenograft mouse models, while the other, a clinical study (2), indicates improved outcome when patients are treated with a combination of both a BRAF^V600E^-specific inhibitor and a MEK inhibitor.

The BRAF^V600E^ mutation is present in ∼50% of human melanomas (4, 5). Prior to its discovery as a causal mutation in melanoma, prognosis for melanoma patients was grim as melanoma is notoriously unresponsive to chemo- and radiotherapies. Following BRAF^V600E^ identification, there was a flurry of activity that culminated in Plexxikon’s development of vemurafenib (Zelboraf) (6), which was approved by the FDA in 2011 and the following year saw approval in Canada and Europe. Moreover, while vemurafenib has been a boon, promoting tumor regression and improved survival, acquired resistance frequently occurs after several months of vemurafenib treatment leading to MAP kinase pathway re-activation (6) that is not blocked by vemurafenib. How was the resistance happening and what can be done to minimize resistance up front?

Now, recent work from the Rosen lab at Memorial Sloan-Kettering Cancer Center has uncovered data that vemurafenib reduces feedback inhibition in BRAF^V600E^-expressing cells such that these cells become sensitized to extracellular growth factors like hepatocyte and epidermal growth factors. Unlike canonical receptor-mediated RAF signaling, which utilizes RAF-RAF dimers, BRAF^V600E^ functions as a monomer that is sensitive to RAF inhibition. Vemurafenib and other BRAF^V600E^ inhibitors like dabrafenib (GlaxoSmithKline, currently in Phase III trials) inhibit the BRAF^V600E^ monomer but are unable to block ligand-mediated RAF-RAF dimers. Thus, in the context of persistent vemurafenib, feedback inhibition wanes permitting extracellular mitogens to bypass vemurafenib-mediated inhibition since they promote RAF-RAF dimers. Importantly, the authors go on to show using four different BRAF^V600E^ melanoma xenograft mouse models that an effective dose of Plexxikon’s vemurafenib analog PLX4720, when combined with a low dose of the MEK inhibitor PD0325901 promotes tumor regression in 25/26 tumors. Either PLX4720 or PD0325901 alone also inhibited tumor growth but less potently that when combined.

Concurrently, in a recent study published in the New England Journal of Medicine, Flaherty and colleagues provide promising data from an open-label trial (2) involving over 400 patients that a combination of the BRAF^V600E^ inhibitor, dabrafenib, and a MEK inhibitor trametinib (GlaxoSmithKline), which is near approval (7), significantly increased progression-free survival when compared to monotherapy. These studies validate the rationale to try to anticipate acquired resistance at the onset of therapy, rather than dealing with it once it has already occurred.

Going forward, how might targeted therapies be best designed to overcome acquired resistance, especially when parallel pathways become activated? Recent work (8) published in Nature from the Cagan and Shokat labs at Mt. Sinai School of Medicine and University of California, San Francisco, CA, USA respectively, suggests a systems-based strategy to do just that. In a *Drosophila* model of Ret-induced multiple endocrine neoplasia, the authors show using a rationally designed single small molecule that balanced inhibition of multiple targets (Raf, Src, and S6K) was the most efficacious especially when the molecule was designed to avoid a so-called “anti-target,” Tor in this case, to minimize toxicity and relief from feedback inhibition. There is cautious optimism that such strategies will have success in the clinic (1).

Finally, these data raise questions for the possible future therapies (9) for the developmental disorders caused by MAP kinase pathway mutations, collectively referred to as the RASopathies, and in particular cardio-facio-cutaneous (CFC) syndrome in which the majority of cases are caused by gain-of-function BRAF mutations.
